# Prebiotic properties of jiaogulan in the context of gut microbiome

**DOI:** 10.1002/fsn3.2701

**Published:** 2022-01-22

**Authors:** Gouxin Huang, Muhammad Yasir, Yilin Zheng, Imran Khan

**Affiliations:** ^1^ Clinical Research Center Shantou Central Hospital Shantou China; ^2^ Special Infectious Agents Unit King Fahd Medical Research Center King Abdulaziz University Jeddah Saudi Arabia; ^3^ State Key Laboratory of Quality Research in Chinese Medicine Macau University of Science and Technology Taipa China

**Keywords:** gut microbiota, *Gynostemma pentaphyllum* (jiaogulan), polysaccharides, saponins

## Abstract

Jiaogulan (*Gynostemma pentaphyllum*) is a traditional Chinese medicinal herb that has been widely used in food and supplemental products. In the last 20 years, extensive research has been conducted to investigate the medicinal prospects of jiaogulan, and in this regard, more than 200 compounds have been isolated with various medicinal properties such as anticancer, anti‐obesity, anti‐inflammation, and antioxidation. In respect of potential benefits, jiaogulan market is likely growing, and various food items comprised of jiaogulan (beverage, sport drinks, cola, beer, tea, bread, and noodles) have been commercialized in the United States of America, China, and other Asian countries. More recently, there has been growing interest in the prebiotic potential of jiaogulan, especially at the interface of the gut microbiota. This review focuses on the prebiotic and therapeutic aspects of saponins and polysaccharides of jiaogulan tea by summarizing the literature on cancer, obesity, antioxidant activity, and immune‐modulatory properties.

## Graphical abstract

This study summarizes the therapeutic and prebiotic properties of various saponins and polysaccharides from jiaogulan. The study also highlights gut microbiota (GM)‐modulating properties of various compounds from jiaogulan. This review highlights the therapeutic effect of jiaogulan on the diversity and composition of GM.

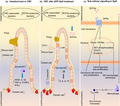

## 1 INTRODUCTION

**FIGURE 1:**
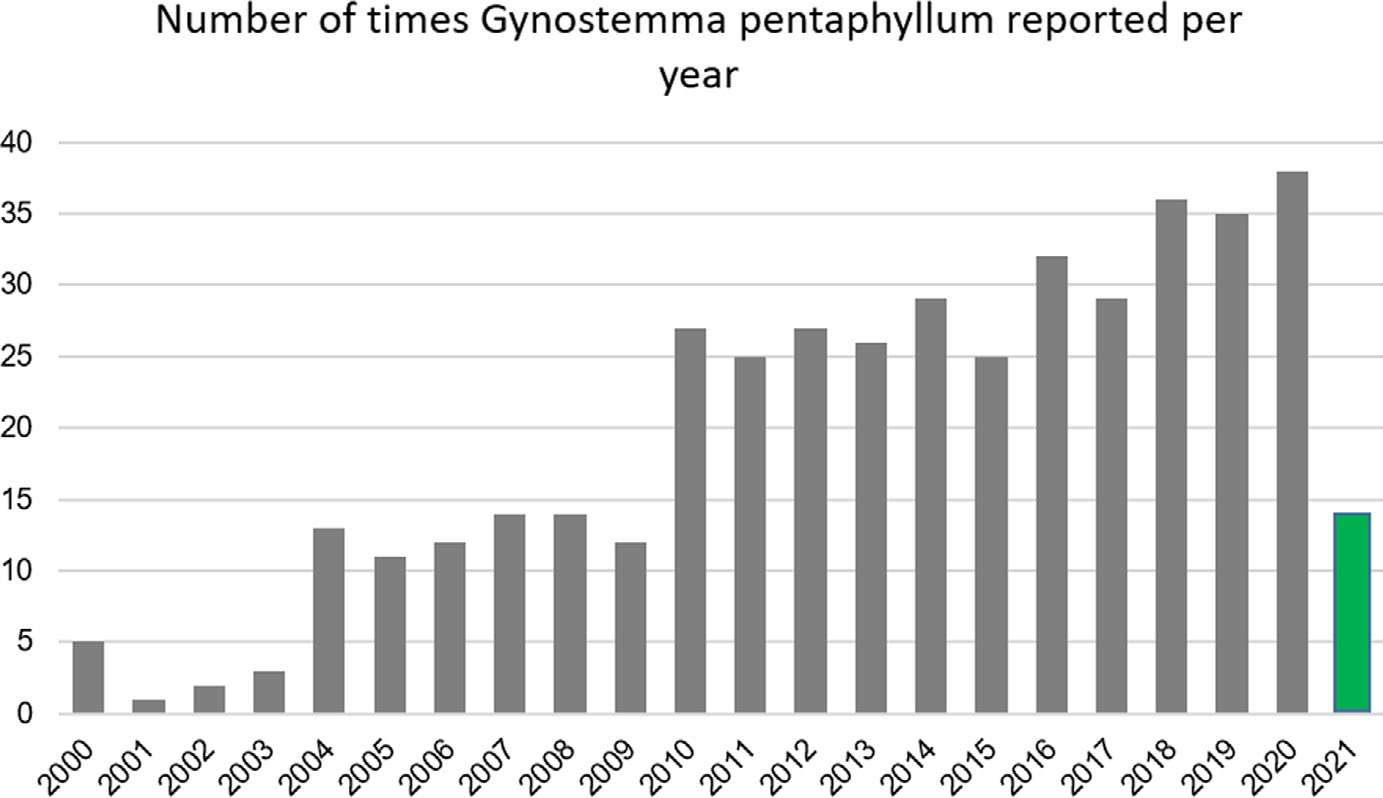
Bar chart presentation of *Gynostemma pentaphyllum* reported in PubMed database. These data were generated by including clinical trials, research articles, review articles, and abstracts. Numbers at the top of the bar show *G*. *pentaphyllum* reported times per year, ranging from 2000 to 2021. The search was conducted on August 4, 2021

*Gynostemma pentaphyllum* Makino (Cucurbitaceae; Gp) is a perennial creeping plant and has been used for herbal tea (called jiaogulan) in China. For centuries, the herbal tea made from the aerial part (including stems and leaves) of Gp has been consumed in China as a general tonic. Today, it is progressively popularizing around the world for lowering serum lipid and cholesterol levels (Chen et al., 1991; Lin et al., 2000). Like green tea, jiaogulan tea also holds anticarcinogenic and antioxidative activities (Lin et al., 2000; Razmovski‐Naumovski et al., 2005). Increasing research interest in Gp is evident from a search of the PubMed database (Figure 1).

Traditionally, jiaogulan has been broadly applied for the treatment of various illnesses, including hepatitis, diabetes, and cardiovascular disease (Li et al., 2019). However, in the last 20 years, extensive research has been conducted to investigate the medicinal prospects of jiaogulan, which has resulted in the discovery of more than 230 compounds with medicinal properties. These compounds have shown a variety of pharmacological properties, including anti‐inflammatory (Cai et al., 2016; Quan & Qian, 2010), antioxidative (Zhao et al., 2014), antiproliferative (Yan, Wang, Niu, et al., 2014), anxiolytic activities (Choi et al., 2013), anti‐cancer (Hou et al., 1991), lipid metabolism regulation (Qin et al., 2012), anti‐diabetes (Gao et al., 2016), and cardiovascular disease treatment (Circosta et al., 2005). Out of 230 compounds, 189 are saponins, also known as gypenosides (Li et al., 2016). Among these gypenosides, 165 have been classified into 12 types based on the nature of aglycone moiety (Lin, 2011). The details of these gypenosides and their pharmacological properties have been discussed elsewhere (Nguyen et al., 2021). Gypenosides possess several therapeutic properties including anticancer and anti‐obesity (Lee et al., 2019; Lu et al., 2010).

Like other basic research, Chinese medicine research is entering into a new paradigm from one‐gene‐one‐phenotype model toward a much sophisticated and complex model, known as omics strategies, that is based on data‐driven untargeted management, diagnosis, and treatment (Cagan et al., 2013; Yoo et al., 2018). Chinese medicine is holistic in nature and it would be impractical to comprehend it with conventional research tools. Therefore, more recently, researchers have started to evaluate the prebiotic potential of jiaogulan at the interface of the gut microbiota (GM).

The pharmacological properties of jiaogulan at the interface of the GM should be evaluated as commensals that play an important role in human physiology. For instance, the human microbiome constitutes about 47% of our body by cell count and encodes 1000 times more genes than our own body genes (Institute for Genome Sciences, 2017; Knight et al., 2017). It is estimated that the human microbiome encodes 2–20 million genes that surpasses the ~20,000 human genes (Knight et al., 2017). These microbial genes are presented to the host for digestion, metabolism, and immune system maturation (Cani, 2009). Targeted intervention to remodel GM composition has shown encouraging results in disease prevention and treatment. Mounting lines of evidence indicate that various bioactive natural products, such as dietary fibers, phenolic compounds, and undigested carbohydrates, can upregulate beneficial intestinal microbes, improve gut homeostasis, and alleviate disease symptoms (Makki et al., 2018).

In this review, we highlight some key findings by evaluating jiaogulan extracts and the therapeutic nature of purified compounds (gypenosides, polysaccharides, and flavonoids) at the interface of the GM. The study particularly focuses on the anticancer, anti‐obesity, and antidiabetic properties of jiaogulan.

## 2 ANTICANCER PROPERTIES

**FIGURE 2:**
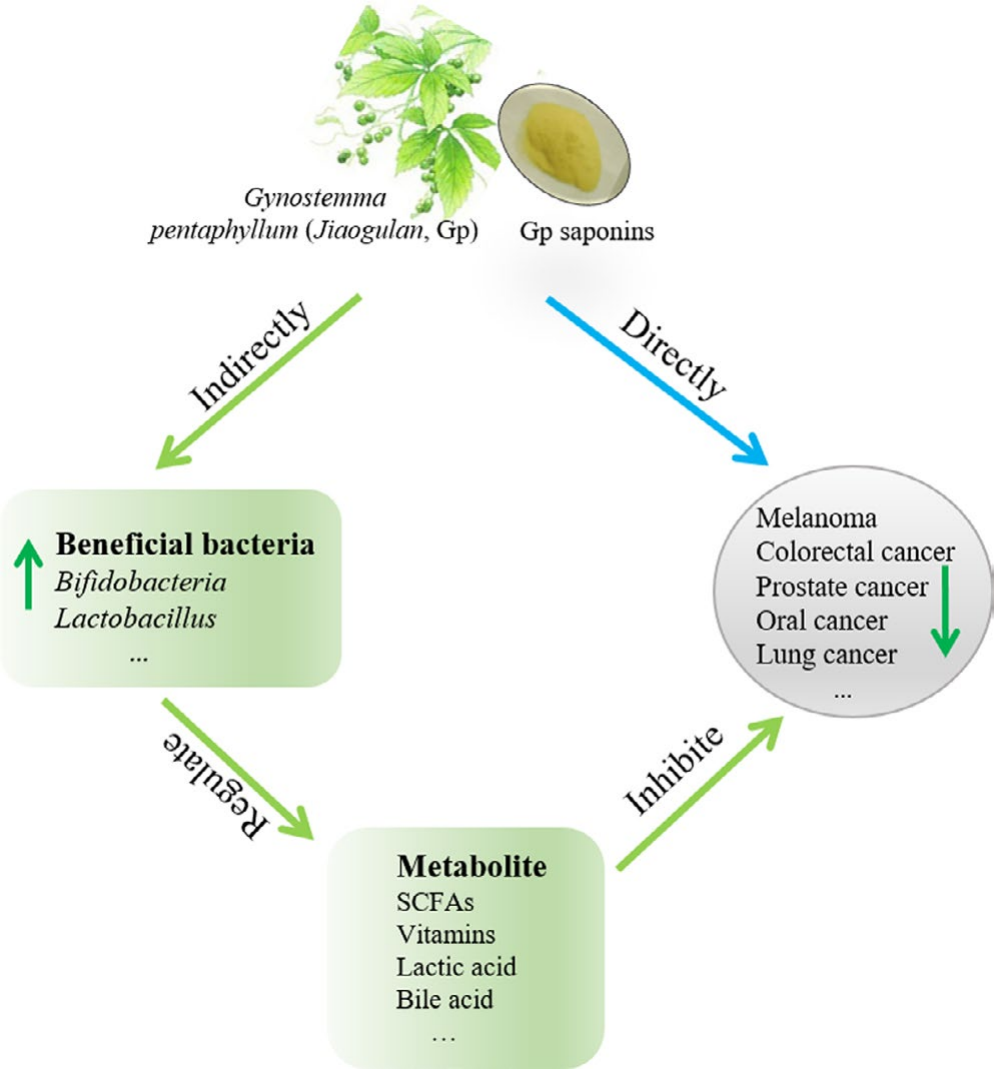
Thematic presentation of the jiaogulan tea's anticancer effects. Through literature review, it is observed that different components of the jiaogulan tea possess anticancer properties that these compounds exert either directly or indirectly. Through indirect approach, jiaogulan tea's component exerts anticancer effects through the interface of the gut microbiota. Here, we summarize that jiaogulan promotes the growth of beneficial bacteria, particularly the short‐chain fatty acid (SCFA) producers. SCFAs eventually exert anticancer properties

Jiaogulan is known to possess potent anticancer abilities. So far, several mechanisms of action have been determined regarding the anticancer activities of jiaogulan, including antioxidant (Li et al., 2015), cell cycle arrest, apoptosis, prevention of invasion and metastasis (Yan, Wang, Niu, et al., 2014), and immunomodulating activities. For instance, *G*. *pentaphyllum* saponin (GpS) was reported for the anticancer properties by upregulating *Prdx1* and *Prdx2* expression and suppressing Ras, RAF/MEK/ERK/STAT, PI3K/AKT/mTOR signaling (Tai et al., 2016). Another in vitro study also showed that GpS revealed the anti‐proliferation effect by arresting cell cycle at the G0/G1 phase, and induced apoptosis of HepG2 cells via death receptor and mitochondrial pathway (Hussain et al., 2020). Yan, Wang, Wang et al. (2014) showed that GpS could significantly upregulate the intracellular ROS level, which induced cell toxicity, apoptosis, and mitochondrial damage in colorectal cancer cells. The anticancer abilities of jiaogulan, both direct and indirect, have been summarized in Figure 2.

### 2.1 Anticancer properties of jiaogulan's saponin

**FIGURE 3:**
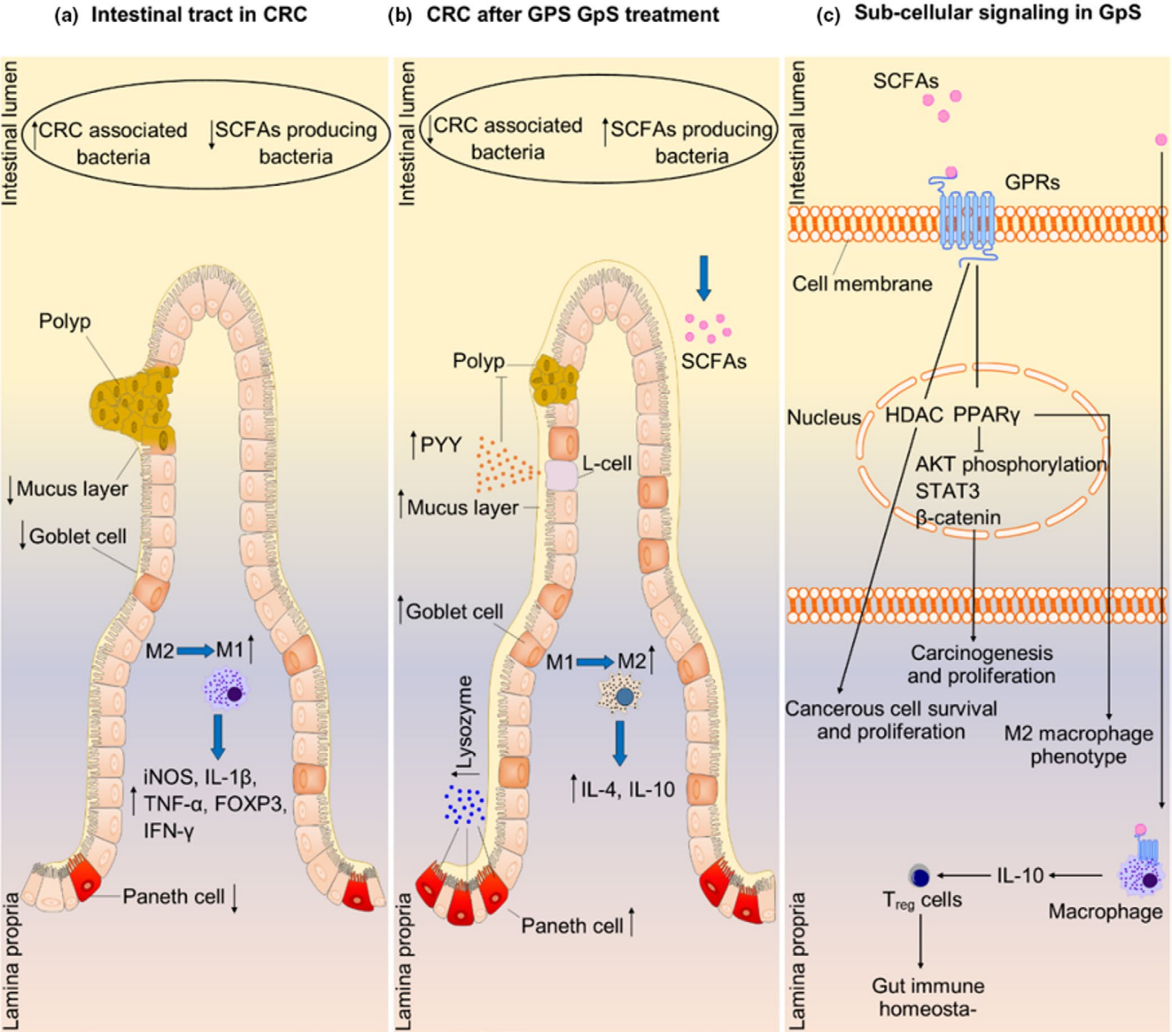
Schematic presentation of GpS' anticancer effects through gut microbiota. (a) In colorectal cancer (CRC), the intestinal track is characterized by polyp formation, imbalanced gut microbiota, reduced mucus layer, suppressed population of goblet and Paneth cells, and inflamed immune milieu. (b) Treated CRC preclinical mouse model with GpS reinstates the inflamed mucosal immunity, promotes goblet and Paneth cell population – that results in mucus layer thickness and higher secretion of lysozyme. Most importantly, the gut microbial composition improves with the prevalence of SCFA producers. (c) At the subcellular level, GpS‐associated increase in SCFAs upregulates fatty acid‐sensing GPCRs that results in the suppression of histone deacetylases and PPARγ, which downstream inhibits PI3K/AKT oncogenic signaling pathways, as well STAT3 and Src. This graph is based on results published by Hsiao's group (Chen et al., 2016; Khan et al., 2019; Liao et al., 2020)

Unlike the in vitro system, saponins are poorly absorbed and have a long residence time in the intestine when tested in preclinical models (Navarro del Hierro et al., 2018). However, through the recent integration of herbal medicine and GM research, the long residence of saponin in the intestine turned out to improve its efficacy. We and several other studies have demonstrated that GpS improves gut microbial composition by promoting the growth of beneficial bacteria and suppressing potential pathogens (Chen et al., 2015, 2016; Huang et al., 2017, 2018; Khan et al., 2019; Shen, Zhong et al., 2020). While evaluating the anticancer effects of GpS in a Apc*^Min/+^* mouse model, GpS displayed a stimulating effect on the abundance of *Lactococcus*, *Bifidobacterium*, *Lactobacillus*, and short‐chain fatty acids (SCFAs) producing bacteria. However, the growth of potential pathogens, for example, *Dysgonomonas* spp., *Helicobacter* spp., sulfate‐reducing bacteria, were suppressed after GpS introduction to mouse gut (Chen et al., 2015, 2016; Huang et al., 2017; Khan et al., 2019; Liao et al., 2020) (Figure 3).

After noticing the stimulating effect of GpS in SCFAs producer in the mouse gut, we proved that GpS could increase the growth of *Bifidobacterium* *animalis*, *Lactobacillus* *casei*, and *Lactobacillus* *reuteri* (Liao et al., 2020). By gavaging *B*. *animalis* and butyrate (separately) to a cancer preclinical mouse model and noticing anticancer effects, we proved that the anticancer effect of GpS is partly through stimulating the growth of beneficial bacteria in the gut (Liao et al., 2020). The anticancer efficacy of GpS could be improved in the presence of polysaccharides. We confirmed the enhanced cancer‐preventive properties of GpS when applied in combination with *Ganoderma lucidum* (Lingzhi) polysaccharides. It was observed that GpS and polysaccharides from *lucidum* can greatly improve the inflamed gut barrier of Apc*^Min/+^* mice by inhibiting polyp formation, changing colonic M1 to M2 macrophages, stopping the oncogenic signaling molecules, and increasing the E‐cadherin/N‐cadherin ratio (Khan et al., 2019).

### 2.2 Anticancer properties of jiaogulan's polysaccharides

In addition to the GpS, GP's polysaccharides (GpP) also be reported for anticancer properties. Polysaccharides are potential prebiotic polymers that have been extensively studied. They promote the growth of certain beneficial bacteria (e.g., *Bifidobacterium*, *Lactobacillus*) and in the large intestine, metabolize into lactic acid and SCFAs that improve host physiology, particularly gastrointestinal health (Azmi et al., 2012; Devillé et al., 2007; Zaporozhets et al., 2014). Accumulating evidence reports that GpP revealed the anticancer effect in vivo and in vitro. The molecular weight, degree of branching, and solubility of GpP are closely related to its anticancer property. Different molecular weights in the range of 10^3^–10^6^ Da have been found in various GpP using different experimental conditions (Ji et al., 2018). A neutral polysaccharide fraction of GP was found to effectively inhibit the solid tumor growth of H22 hepatocarcinoma transplanted in ICR mice (Liu et al., 2014). Another study showed that GpP improved the proportion and mitochondrial level of T cells, and promoted the secretion of IL‐2 and IFN‐γ in the ascites of mice (He et al., 2020). Yu et al. (2020) reported that a novel acidic polysaccharide from GP exhibited significant apoptotic characteristics, such as cell shrinkage, decreased cell adherence, and the appearance of apoptotic bodies in SPC‐A‐1 and MGC‐803 cells. Chen et al. (2011) isolated a novel GpP and synthesized four sulfated polysaccharides from this GpP using the chlorosulfonic acid method. The results showed that the polysaccharides significantly inhibited the growth of HepG2 cells and Hela cells.

### 2.3 Anticancer properties of jiaogulan's flavonoid

Flavonoid is a main polyphenolic compound that is widely found in herbal medicines. Flavonoid is also a major constituent of GP, and reveals certain bioactivities, especially anticancer and antioxidant effects. It has been established the flavonoid from GP (GpF) and GpS could equally suppress the growth of prostate cancer PC‐3 cells, with IC_50_ values of 39.3 and 33.3 μg/ml, respectively. These two GP fractions induced cell cycle arrest at both S and G2/M phases as well as apoptosis (Cheng et al., 2011). Another research reported that GpF induced apoptosis and concomitantly altered the balance of BCL‐2 and BAX expression as well as caspase‐3 expression in both A549 and H469 lung cancer cell lines. However, the authors found that GpF induced cell cycle arrest at both S and G2/M phases and regulated cellular proteins cyclin A, B, p53, and p21 expression in A549, but not H460 (Tsui et al., 2014). Lin et al. (2019) isolated four flavonoids from GP using chromatography and found that the flavonoids could act against AAPH‐induced oxidative damage in LLC‐PK1 cells by suppressing the increase in MDA, and the decrease in SOD and glutathione. Wang, Yang, et al. (2018) showed that GpF exerted antioxidant effect on A549 with H₂O₂‐induced oxidative stress through increasing SOD, GSH, and HO‐1 expression and simultaneously decreasing ROS and MDA expression. Jang et al. (2016) isolated eight flavonoids from GP, including a novel compound, and evaluated the antioxidative effect by the DPPH radical scavenging assay. The results showed that rutin possessed the strongest antioxidative property.

## 3 HEPATOPROTECTIVE EFFECT

Lipopolysaccharide (LPS) and Toll‐like receptor 4 (TLR4) are significantly increased during the progression of nonalcoholic fatty liver disease (NAFLD). A report showed that GpS improved NAFLD induced by high‐fat diet induced through regulating LPS/TLR4 signaling pathway (Shen, Wang et al., 2020). Hong et al. investigated the underlying mechanisms of GP and its derived compounds on protecting NAFLD through network pharmacology prediction. The authors claimed that GpS, especially gypenoside XL, could target peroxisome proliferator‐activated receptor alpha (PPARα), the expression of which was downregulated in alcoholic fatty liver disease and NAFLD patients in the liver. Further research proved that gypenoside XL could upregulate the expression of acyl‐CoA oxidase and carnitine palmitoyltransferase‐1, which contributed to the anti‐NAFLD effect (Hong et al., 2018). Other related reports showed that GpS protected against NAFLD progression by upregulating the expression of PPARα and downregulating the inflammatory cytokines, oxidative stress indices, and de novo lipogenesis (Gou et al., 2016; He et al., 2015; Qin et al., 2012). Bae et al. (2018) reported that gypenoside UL4 enriched in GP extract exerted the hepatoprotective effect on diet‐induced NAFLD through increasing levels of sirtuin 6 and phase 2 antioxidant enzymes in vivo and in vitro. A recent study showed that GpS could change the GM composition of NAFLD mice to alleviate disease progression. The results showed that GpS reduced the ratio of Firmicutes to Bacteroidetes, elevated GM diversity, and decreased the relative abundance of *Fissicatena* and *Akkermansia*, which are enriched in high‐fat and high‐cholesterol‐induced NAFLD mice (Huang et al., 2019). Similar research also showed that GpS alleviated NAFLD by maintaining the gut barrier and reversing gut dysbiosis in a high‐fat diet‐induced NAFLD rat model. Results showed that GpS reduced the ratio of Firmicutes to Bacteroidetes; meanwhile, GpS enriched the abundance of beneficial bacteria (*Lactococcus* spp.) and inhibited potential pathogens (Shen, Zhong, et al., 2020).

## 4 ANTI‐OBESITY EFFECT

AMPK is an intracellular energy sensor and regulates the whole‐body and cellular energy balance in response to energy demand and supply. Nguyen et al. (2011) demonstrated that dammarane‐type glucosides from GP were the potential activator of AMPK. Further study from this research team also showed that GP enriched with saponins could improve obesity in *ob/ob* mice by activating AMPK (Gauhar et al., 2012). Lee et al. demonstrated that GP extract enriched with gypenosides could reduce serum levels of triglyceride, total cholesterol, and LDL‐cholesterol and display the anti‐obesity effect in HFD‐induced obesity. The research mechanism showed that GpS could increase AMPK activation and suppress adipogenesis by decreasing CCAAT/enhancer‐binding protein‐α (C/EBPα), PPARγ, sterol regulatory element‐binding protein‐1c (SREBP1c), PPARγ coactivator‐1α, fatty acid synthase, adipocyte protein 2, and sirtuin 1 (Lee et al., 2019). Inhibiting pancreatic lipase activity is considered one of the treatment strategies for obesity. Previous reports showed that GpS could inhibit pancreatic lipase activity and possibly possess anti‐obesity effect (Bai et al., 2010; Su et al., 2016). Liu et al. found that GpS significantly reduced body weight, plasma total cholesterol, and homeostasis model assessment‐estimated insulin resistance index in a HDF‐induced obese mice model. The authors also showed that GpS could increase brown adipocyte tissue activity and white adipose tissue browning. The gene expression involved in mitochondrial activity and fatty acid β‐oxidation were also increased in both brown and white adipocyte tissues. Moreover, GpS decreased the ratio of Firmicutes to Bacteroidetes, and increased *Akkermansia muciniphila* abundance in the GM (Liu et al., 2017).

## 5 ANTIDIABETIC EFFECT

*Gynostemma pentaphyllum* saponin has been reported for hypoglycemic properties by enhancing the Nrf2 signaling pathway in streptozotocin‐induced diabetic rats (Gao et al., 2016). GP containing standardized gypenosides significantly elevated the plasma insulin concentration and profoundly affected the intraperitoneal insulin tolerance test compared with the control group (Wang, Ha, et al., 2018). In the streptozotocin‐induced diabetic rat model, GpS showed the hypoglycemic effect through enhancing the Nrf2 signaling pathway. Results also showed that GpS increased the level of insulin in the blood, as well as increased SOD and GSH‐px activities (Gao et al., 2016). Norberg et al. (2004) isolated a novel gypenoside from GpS, which was named phanoside, and the results showed that phanoside and its stereoisomers could significantly stimulate insulin secretion. Wang, Ha, et al. (2018) found that two compounds from gypenosides could significantly enhance 2‐deoxy‐2‐[(7‐nitro‐2,1,3‐benzoxadiazol‐4‐yl)amino]‐D‐glucose (2‐NBDG) uptake and glucose transporter 4 (GLUT4) translocation via activating the AMPK and acetyl‐CoA carboxylase signaling pathway. Recently, in a research profiling and screening the active compounds of GP in the diabetic rat model, 27 dammarane‐type triterpenoids were characterized by mass spectrometry and NMR spectroscopy. One of these triterpenoids showed glucose‐dependent insulin secretion activity (Lundqvist et al., 2019). These studies provided the potential candidates for the development of antidiabetic agents of GpS.

Except for the mentioned effects, GpS also possesses other bioactive properties. Research showed that GpS possessed the anti‐fatigue property for exercise‐induced fatigue. GpS could extend the swimming time for the mice, effectively delay the lowering of glucose in the blood, and prevent the increase in lactate (Ding et al., 2010). Aktan et al. (2003) found that GpS could suppress NO synthesis in murine macrophages by inhibiting iNOS enzymatic activity and attenuating NF‐κB‐mediated iNOS protein expression. Tsang et al. (2019) reported that GpS induced melanogenesis and activated cAMP/PKA and Wnt/β‐catenin signaling pathways in both B16 and B16F10 cells. Yang et al. (2013) found that two new saponins from Gp could inhibit lipopolysaccharide (LPS)‐induced IL‐1β, IL‐6, and COX‐2 mRNA expression in RAW 264.7 which showed a prominently anti‐inflammatory effect.

## 6 ANTIOXIDANT EFFECT

Li et al. (2015) isolated three acid polysaccharides from GP—GPA1 (19.6 kDa), GPA2 (10.6 kDa), and GPA3 (6.7 kDa)—that displayed the antioxidant effect through scavenging 1,1‐diphenyl‐2‐picrylhydrazyl (DPPH) radical and hydroxyl radical, chelating ferrous ion, and reducing ferric ion. GpP increased the scavenging activity of DPPH, hydroxyl radical, superoxide anion, and ABTS radical in vitro (Li et al., 2015). Furthermore, an animal experiment showed that GpP could enhance SOD, CAT, GSH‐Px activities, and decrease MDA activity (Wang et al., 2020). Yu et al. (2020) isolated a novel acid polysaccharide from Gp, and antioxidant assays showed that this GpP could scavenge superoxide radical, ABTS, and DPPH radicals. Chi et al. (2012) showed that GpP significantly prolonged the exercise time to exhaustion of mice, and increased the glycogen level and antioxidant enzymatic activity in the skeletal muscle.

## 7 IMMUNOMODULATION EFFECT

Mounting evidence indicated that polysaccharides from Chinese herbal medicines usually act as an immunomodulator that provides benefits for the host (Chen et al., 2020; Gan et al., 2004; Khan et al., 2019; Xu et al., 2011; Zhao et al., 2010). GpP activated macrophage phagocytosis and NK cells, and exhibited activity on none or Con A/LPS‐stimulated splenocytes in C57BL/6 mice. GpP also increased CD4^+^ lymphocyte quantitation and the ratio of CD4^+^/CD8^+^, and increased IL‐2 secretion in serum and spleen in immunosuppressed mice (Shang et al., 2016). Ren et al. reported that the acid polysaccharide fraction from Gp could markedly promote the secretion of NO, TNF‐α, IL‐1β, and IL‐6 in murine macrophage RAW264.7. The authors claimed that MAPK, PI3K/Akt, and NF‐κB signaling pathways were involved in these GpP‐induced macrophage activations (Ren et al., 2019). The neutral polysaccharide fraction from GP could modulate the activity of NK cells and cytotoxic T lymphocytes besides increasing the secretion of IL‐2, TNF‐α, and IFN‐γ in tumor‐bearing mice (Liu et al., 2014).

The hepatoprotective effect of GpP was proved by decreasing serum ALT and AST levels, as well as the hepatocyte MDA content and hepatocyte necrosis in the liver‐injured animal model (Song et al., 2013; Zhang, 2013). GpP possessed hypoglycemic and hypolipidemic effects in a streptozotocin‐induced type 2 diabetes rat model (Du et al., 2011). Jia et al. (2015) investigated the neuroprotective effect of GpP and found that GpP could be effective against Aβ (25–35)‐induced neurotoxicity in PC12 cells by inhibiting oxidative stress and suppressing the mitochondrial apoptotic pathway (Jia et al., 2015). Moreover, an associated research also showed that treatment with GpP could markedly increase the exhaustive exercise time of mice through scavenging excessive ROS produced during the exercise regimen (Chi et al., 2012).

## 8 CONCLUSION

This study summarized the therapeutic and prebiotic properties of various saponins and polysaccharides from jiaogulan. The study also highlighted GM‐modulating properties of various compounds from jiaogulan. This review further highlighted the therapeutic effect of jiaogulan on the diversity and composition of the GM.

## ACKNOWLEDEGMENT

We thanks all the authors' contribution to this work. Thanks for the suggestion from Prof. Li Qingnan from Shantou central hospital. Thanks for the foundation of The Hospital Incubation Project from Shantou Central Hospital (2020‐2022).

## CONFLICT OF INTEREST

The authors declare no competing financial interest.

## ETHICAL APPROVAL

Not applicable.

## Data Availability

Data sharing is not applicable to this article because no new data were created in this study.
